# Transactions of the Medical Association of the State of Alabama, 1902

**Published:** 1903-09

**Authors:** 


Transactions of the Medical Association of the State of Alabama, 1902.

The State Medical Association of Alabama is really the State Board of Health, and considerable business pertaining to health affairs is recorded in the transactions. The volume has over 480 pages, and contains a full quota of interesting papers and their discussions. T. J. B.

